# *EXERGAMES* AS A TOOL FOR THE ACQUISITION AND DEVELOPMENT OF MOTOR SKILLS AND ABILITIES: A SYSTEMATIC REVIEW

**DOI:** 10.1590/1984-0462/;2017;35;4;00013

**Published:** 2017-09-21

**Authors:** Pâmella de Medeiros, Renata Capistrano, Marcela Almeida Zequinão, Siomara Aparecida da Silva, Thais Silva Beltrame, Fernando Luiz Cardoso

**Affiliations:** a Universidade do Estado de Santa Catarina, Florianópolis, SC, Brasil.; b Universidade do Minho, Braga, Portugal.; c Universidade Federal de Ouro Preto, Ouro Preto, MG, Brasil.

**Keywords:** Motor skills, Physical education, Literature review, Habilidade motora, Educação física, Revisão

## Abstract

**Objective:**

To analyze the literature on the effectiveness of *exergames* in physical education classes and in the acquisition and development of motor skills and abilities.

**Data source:**

The analyses were carried out by two independent evaluators, limited to English and Portuguese, in four databases: Web of Science, Science Direct, Scopus and PubMed, without restrictions related with year. The keywords used were: “*Exergames* and motor learning and motor skill” and “*Exergames* and motor skill and physical education”. The inclusion criteria were: articles that evaluated the effectiveness of *exergames* in physical education classes regarding the acquisition and development of motor skills. The following were excluded: books, theses and dissertations; repetitions; articles published in proceedings and conference summaries; and studies with sick children and/or use of the tool for rehabilitation purposes.

**Data synthesis:**

96 publications were found, and 8 studies were selected for a final review. The quality of the articles was evaluated using the Strengthening the Reporting of Observational Studies in Epidemiology (STROBE) scale and the Physiotherapy Evidence Database (PEDro) scale. Evidence was found on the recurring positive effects of *exergames* in both motor skills acquisition and motor skills development.

**Conclusions:**

*Exergames*, when used in a conscious manner - so as to not completely replace sports and other recreational activities -, incorporate good strategies for parents and physical education teachers in motivating children and adolescents to practice physical exercise.

## RESUMO

**Objetivo:**

Analisar a literatura quanto à eficácia da utilização dos *exergames* nas aulas de Educação Física e na aquisição e desenvolvimento de habilidades e capacidades motoras.

**Fontes de dados:**

As buscas dos estudos foram realizadas por duas avaliadoras de forma independente, limitadas às línguas inglesa e portuguesa, em quatro bases de dados: Web of Science, Science Direct, Scopus e PubMed, sem restrições de ano. As palavras-chave utilizadas foram: *“Exergames and motor learning and skill motor” e “Exergames and skill motor and physical education”.* Adotaram-se como critérios de inclusão: artigos que avaliaram a eficácia da utilização dos *exergames* nas aulas de Educação Física e na aquisição e desenvolvimento de habilidades e capacidades motoras. Excluíram-se da análise: livros, teses e dissertações; artigos repetidos; conferências, artigos publicados em anais e resumos de congressos; amostras com patologias e/ou com fins de reabilitação.

**Síntese dos dados:**

Foram encontradas 96 publicações. Após a aplicação dos critérios de exclusão, oito artigos foram selecionados. A qualidade dos artigos foi avaliada pelas escalas *Strengthening the Reporting of Observational Studies in Epidemiology* (STROBE) e *The Physiotherapy Evidence Database* (PEDro). Foi possível verificar evidências sobre os efeitos positivos da utilização dos *exergames* tanto na aquisição de habilidades motoras quanto no desenvolvimento de capacidades motoras.

**Conclusões:**

Os *exergames,* quando utilizados de forma consciente - de modo a não substituir completamente os esportes e outras atividades lúdicas -, representam boas estratégias para pais e professores de Educação Física na motivação à prática do exercício físico.

## INTRODUCTION

As an indispensable factor for success in sports activities, games and other physical activities, basic motor skills in childhood are determinant for a healthy and active lifestyle.^1^ On the other hand, physical inactivity in childhood may result in the inability to acquire and develop motor skills and abilities, which leads to posterior deficit in learning and in the perfection of specialized motor abilities.^2^ Some variables make it difficult to practice physical activity in school environments, such as: limited time, large number of students per class and lack of adequate spaces. Besides, throughout the years there has been a change in the behavior of children, leading to the removal of games that involve the movement of several body segments, and to the approximation with technology and entertainment using a screen. Facing this phenomenon, new strategies are required to keep the children motivated for the practice of physical activity.^3^

Aiming at allying technology and physical activity, the active games came up - or exergames, name given to the technologies that require the whole body to move, combining physical exercises and videogames.^4^ These tools convert the real movements to the virtual environment, allowing the users to be more active^5^, practicing virtual sports, fitness exercises and/or other ludic and interactive physical activities, using movements that are similar to real life tasks.^6^ The *exergames* are different from sedentary videogames^7^ due to the physical effort and motor skills and abilities required during the games.^5^

The insertion of exergames in the daily life may help children and adolescents to reach the recommended levels of physical activity, and, probably, have a positive impactive on the lives of children, since this is a useful way to acquire and develop motor skills and abilities.^4^ ^,^ ^8^ ^,^ ^9^ ^,^ ^10^ Even if exergames are a reality in the lives of children and adolescents - and some researchers have been studying their applicability for the motor performance -, identifying evidence in the scientific literature that indicates the successful or little efficient initiatives in relation to their use for the acquisition and development of motor skills and abilities is essential to formulate new proposals for its broad application in the school context.

In this context, the objective of this study was to analyze the literature as to the efficacy of the use of exergames in Physical Education classes and in the acquisition and development of motor skills and abilities.

## METHOD

A systematic review was conducted according to criteria in the Prisma declaration.^11^

The search was conducted in May, 2015, and was limited to texts in English and in Portuguese, only in scientific publications, without year-related restrictions. Inclusion criteria were: articles that assessed the efficacy of the use of exergames in Physical Education classes and in the acquisition and development of motor skills and abilities; free-access studies.

On the other hand, exclusion criteria were: books, chapters of books, theses and dissertations; repeated scientific articles, conferences, articles published in annals and abstracts of congresses; samples with pathologies and/or with rehabilitation purposes.

The search for productions referring to the theme were conducted by two independente evaluators, without year restrictions, and in four databases: Web of Science (https://isiknowledge.com), Science Direct (http://www.sciencedirect.com), Scopus (http://www.scopus.com) and PubMed (http://www.pubmed.com). The following terms were crossed for the search: “*Exergames* e aprendizagem motora e habilidade motora”, “*Exergames* e habilidade motora e educação física”, “*Exergames AND motor learning AND skill motor*”, *“Exergames AND skill motor AND physical education”.*

At first, the titles related with the subject were demonstrated. Then, the studies were selected by reading the titles, using the inclusion and exclusion criteria that were previously established. Afterwards, there was a detailed reading of the abstracts, and then, the articles whose abstracts did not include the aforementioned eligibility criteria were excluded. Finally, the remaining texts were assessed in full. In the study, there was also an analysis of the references of the selected manuscripts.^12^

The quality of the studies was evaluated independently by two reviewers/authors, by the recommendations of two instruments: The Physiotherapy Evidence Database (PEDro), recommended for the evaluation of intervention studies, and the Strengthening the Reporting of Observational Studies in Epidemiology (STROBE), recommended for the evaluation of observational studies.

The PEDro scale,^13^ based on the Delphi list^14^ and translated to Portuguese in 2009, presents 11 items that assess the methodological quality of random clinical trials, observing two aspects of the study: the internal validity and the presence of sufficient statistical information to make it interpretable. Only 10 of the 11 criteria assessed receive scores, so the first question had no classification. Each criterion is scored according to its manifestation in the study assessed: in its presence, one point is attributed; in its absence, there is no score. The final score, presented in Table 1, is obtained by the sum of all the questions that had positive answers. Studies whose score is lower than three are considered with low methodological quality.^15^ According to Verhagen et al.^14^, for the analyses to be classified as high quality, the intervention studies should have scores higher than 50% in relation to the maximum classification. Therefore, for this review, all randomized studies with scores higher than or equal to five were considered to have high methodological quality.

**Table 1:**
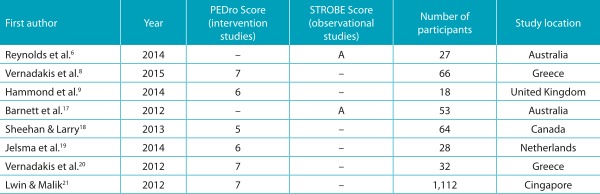
Articles included in the review.

PEDro: *The Physiotherapy Evidence Database*; STROBE: *Strengthening the Reporting of Observational Studies in Epidemiology.*

Besides the PEDro scale, the STROBE scale was also used.^16^ Each one of the 22 criteria received scores of 0 to 1. After the evaluation of the criteria, each article received grades from 0 to 22 from each reviser. The final grade was obtained by the average of the grades, and the variation of grades between the revisers was not higher than 1. The scores of the instruments were turned into percentage rates in order to evaluate the quality of the articles better. Three categories for the evaluation of quality were established: when the study fulfilled more than 80% of the criteria established; when 50 to 80% of the criteria were met; and when less than 50% of the criteria were fulfilled.

 As an example, in the STROBE scale, the articles scored considering the details in the theoretical references, the reasons to execute the study and the information about the sampling size. In the PEDro scale, the intervention studies scored, for instance, considering the eligibility criteria that were specified and the subjects that were randomly distributed in groups. Considering the reduced number of articles selected, these analyses aimed at discussing factors related with the quality of the articles, instead of constituting an exclusion criterion.

## RESULTS

**Figure 1:**
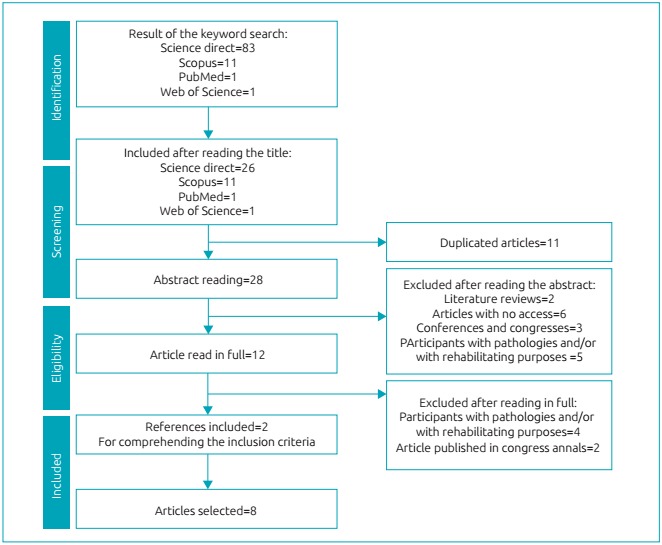
Flowchart of the articles found .

**Chart 1:**
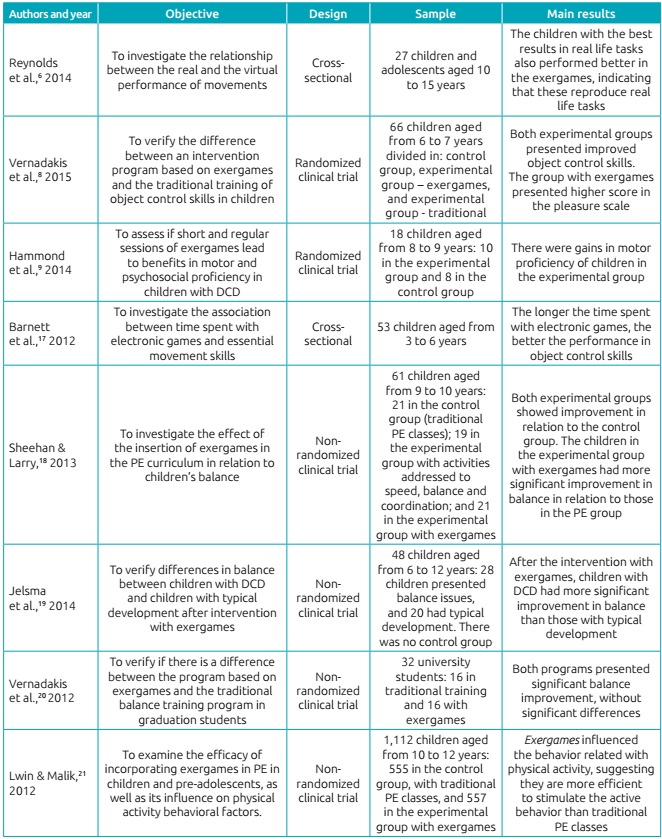
Presentation of the studies selected according to objectives, methods and main results.

PE: Physical Education; DCD: developmental coordination disorder.

We found 96 publications in the databases Science Direct (n=83), Scopus (n=11), PubMed (n=1), and Web of Science (n=1). After the exclusion for duplicity (n=11), 28 articles were selected for the abstract reading. After this stage, 16 articles were excluded; 2 for being literature reviews, 6 for not presenting free access to the study in full, 5 for assessing children with pathologies and/or with rehabilitating purposes, and 3 for being published in conferences and congresses. Twelve studies were left for the full reading, of which 6 were excluded: 4 for assessing children with pathologies and/or with rehabilitating purposes, and 2 for being published in congress annals. Throughout the reading, two relevant references were included in the analysis for approaching the inclusion criteria. Therefore, eight articles were selected for the final review, as shown in Figure 1. Chart 1 presents the main information about the eight articles selected for the final review.

Table 1 demonstrates the data about the methodological quality of the articles. All studies were published from 2012 to 2015, in journals classified with an “A” in Qualis Brasil. Regarding the scores obtained in the PEDro scale, for intervention studies, and in the STROBE scale, for observational studies, the eight articles were considered to have high methodological quality.

## DISCUSSION

In the evaluation of the quality of the studies, most of them met the criteria to fit category “A” in the STROBE classification (more than 80% of the criteria were fulfilled), and the intervention studies presented scores higher than 5 in the PEDro evaluation, which values the credibility of the evidence produced in the analyzed studies.

In the literature selected, four studies were directly related with the acquisition of motor skills. In the first one, Hammond et al.^9^ evaluated the possible benefits of exergames in the motor proficiency of children with developmental coordination disorder (DCD). The authors conducted a motor intervention with Wii Fit for one month, 3 times a week, with 18 children distributed in 2 groups: control (n=8), and experimental (n=10). After the motor evaluation using the BOT-2 battery, before and after the interventions, it was possible to observe significant gain in motor skills, indicating that exergames are simple instruments, able to improve the children’s motor performance.

In the second study, a cross-sectional study by Reynolds et al.^6^ investigated the relationship between the performance of the real movement and the performance of the virtual movement in the exergame, including 27 children aged from 10 to 15 years. The results showed that the children with better scores in real life tasks, verified by the MABC-2 battery, also performed better in the Kinect Sports game (athletics modality). The authors concluded that the virtual games reproduce real life tasks, therefore being a useful source of intervention for the acquisition of motor skills.

In the third study, also cross-sectional, Barnett et al.^17^ analyzed the associations between the time spent on electronic games and the fundamental motor skills in 53 children aged from 3 to 6 years. The authors verified that the longer the time spent playing videogames, the better the performance in object control skills. However, they conclude that longitudinal and experimental studies are necessary to determine if the games actually improve these abilities.

In the fourth study, Vernadakis et al.^8^ analyzed the evidence from the previous study. The authors investigated if there are differences between an intervention program based on an exergame and a traditional training of object control skills in elementary school children. Sixty-six children took part, and 22 were in the control group, whereas 22 formed the experimental group and 22 were involved in the traditional approaches. The intervention was divided in 8 sessions of 30 minutes each, twice a week. Three evaluations were conducted with the TGMD-2 battery, before and after the interventions and one month later, to verify learning retention. The authors observed improvement in the object control skills in both experimental groups, suggesting that the use of the Kinect for motor interventions is a valuable, viable and pleasant approach.

The results found in these four articles can be explained by the fact that the exergames require motor skills and abilities in a variety of cognitive and motor tasks, with a wide range of sensory feedback.^22^ The latter is an essential element in the process of teaching-learning of motor skills, besides being essential to detect and correct errors aiming at the good performance in the motor learning.^23^

Exergames are tools that attract and motivate children for the practice of physical activities. The studies by Jelsma et al.^19^ and Vernadakis et al*.* ^20^ show that interventions with exergames are more attractive than the traditional approaches, and, therefore, might be more efficient in the development of motivation for physical activity and in the assistance to acquire motor skills, since they incorporate essential learning elements.^8^ ^,^ ^24^ Besides, the active games do not just offer the practice of motor skills in real time, but also the opportunity to be involved in intense movements related to interests of daily life.^24^ ^,^ ^25^ ^,^ ^26^

Of the four articles left from the search, three used exergames as an intervention tool to improve the balance in children^18^ ^,^ ^19^ and Young adults.^20^ Interventions occurred from six to ten weeks, and they all had positive results, showing the efficacy of the use of exergames. The authors conclude that the motor intervention with the use of the exergame should be considered as a tool to improve the balance and develop motor skills, so the exergame would be a practical resource for school Physical Education. Such results can be a result of physical effort, of the greater energy expenditure and of the motor skills required by the game, such as balance, resistance, upper and lower limb coordination, speed, strength and flexibility,^5^ ^,^ ^27^ contradicting the idea of a sedentary lifestyle, passivity and physical inactivity of the videogame player.^28^ Besides, the exergames stimulate rhythm, cooperation, creativity and the sports spirit, also developing the motor repertoire.^26^ For Sousa,^29^ the *exergames* lead to motor learning and to the improvement of aspects related with health and physical shape.

Finally, the last article selected examined the efficiency of the incorporation of the exergame in Physical Education for children and pre-adolescents, as well as its influence in physical activity behavioral factors.^21^ Lwin & Malik conducted a motor intervention based on exergames during Physical Education lessons for 6 weeks, with 557 children aged between 10 and 12 years. The results show that the exergame had a significant influence on physical activity behaviors, and the authors concluded that its incorporation may be an efficient alternative to reinforce such behaviors in regular Physical Education classes.^21^

This resource is already used in the state of West Virginia, in the United States, where the schools are betting on exergames during Physical Education classes as a more encouraging way to practice physical exercises. In terms of benefits to health, the exergames have the power to help the children to adopt a healthy lifestyle and to become physically active for life, besides being appropriate to reinforce the levels of physical activity.^9^ However, it is important to emphasize that active games do not replace sports and other forms of physical activity completely.^30^ ^.^ ^31^ Although the movements simulate activities of daily life, the performance of the skill is not identical in virtual environments.^30^ Besides, the games should be ministered in a controlled manner, because of the role of school Physical Education overcomes the limits of the practice of physical activity, since it also plays the role of developing social, affective, emotional and personal relationships. However, the exergames should be seen as a movement innovation, expanding its possibilities^27^ and subsidizing Physical Education classes for being a more ludic and more attractive tool nowadays.

The theme of exergames is very recent in the literature. Despite having its efficacy proven, its use is not common in the school context, so only few schools know the real functionality and the benefits of these games in the motor performance of children. Therefore, this study aimed at bringing to light studies that have already been published on the subject, showing that these results are positive and that the exergames can be used as an alternative work tool in Physical Education classes. However, the review also presents some limitations, such as: use of articles published only in English and in Portuguese, which may have caused the exclusion of studies that approached the theme in other languages; use of only four data bases, preventing the generalization of the results to all scientific publications about the subject; and the exclusion of full articles without free access to researchers, which may have limited the interpretation of results.

## CONCLUSION

The analysis of the results found good evidence about the positive effects of the use of exergames in motor skills and abilities. Facing the urban restrictions that have been taking place all over the world, bringing limitations to childhood motor opportunities, the exergames, when used properly - not completely replacing sports and other ludic activities -, represent good strategies for parents and Physical Education teachers. This tool can help and motivate the practice of physical exercises in domestic and school environments, in order to facilitate the acquisition and improvement of skills, as well as the development of motor skills in a more fun manner for the children.

This review can stimulate the current educators and researchers in the field of motor development and motor learning to be more interested in these popular digital resources. Therefore, we will have one more tool at hand to increase digital inclusion in the current methodological school resources, encouraging physical practices and healthy life habits. Likewise, we can stimulate educational researchers to develop research projects of intervention that continue to study the effects of its use in detail.
